# Fabrication of a 3D Corneal Model Using Collagen Bioink and Human Corneal Stromal Cells

**DOI:** 10.3390/jfb16040118

**Published:** 2025-03-28

**Authors:** Alexander J. Choi, Brenna S. Hefley, Hannah A. Strobel, Sarah M. Moss, James B. Hoying, Sarah E. Nicholas, Shadi Moshayedi, Jayoung Kim, Dimitrios Karamichos

**Affiliations:** 1 North Texas Eye Research Institute, University of North Texas Health Science Center, 3430 Camp Bowie Blvd, Fort Worth, TX 76107, USA; alexander.choi@unthsc.edu (A.J.C.); brenna.hefley@unthsc.edu (B.S.H.); sarah.nicholas@unthsc.edu (S.E.N.); shadi.moshayedi@unthsc.edu (S.M.); jayoung.kim@unthsc.edu (J.K.); 2 Department of Pharmaceutical Sciences, University of North Texas Health Science Center, 3500 Camp Bowie Blvd, Fort Worth, TX 76107, USA; 3 Advanced Solutions Life Sciences, 500 N Commercial St., Manchester, NH 03101, USA; hannah.strobel@advancedsolutions.com (H.A.S.); sarah.moss@advancedsolutions.com (S.M.M.); jhoying@advancedsolutions.com (J.B.H.); 4 Department of Pharmacology and Neuroscience, University of North Texas Health Science Center, 3500 Camp Bowie Blvd, Fort Worth, TX 76107, USA

**Keywords:** corneal bioprinting, cornea, bioengineering, bioink, cornea, corneal fibrosis, ECM

## Abstract

Corneal transplantation remains a critical treatment option for individuals with corneal disorders, but it faces challenges such as rejection, high associated medical costs, and donor scarcity. A promising alternative for corneal replacement involves fabricating artificial cornea from a patient’s own cells. Our study aimed to leverage bioprinting to develop a corneal model using human corneal stromal cells embedded in a collagen-based bioink. We generated both cellular and acellular collagen I (COL I) constructs. Cellular constructs were cultured for up to 4 weeks, and gene expression analysis was performed to assess extracellular matrix (ECM) remodeling and fibrotic markers. Our results demonstrated a significant decrease in the expression of COL I, collagen III (COL III), vimentin (VIM), and vinculin (VCL), indicating a dynamic remodeling process towards a more physiologically relevant corneal ECM. Overall, our study provides a foundational framework for developing customizable, corneal replacements using bioprinting technology. Further research is necessary to optimize the bioink composition and evaluate the functional and biomechanical properties of these bioengineered corneas.

## 1. Introduction

Over 80% of the information processed by the brain comes through vision [1]. The visual system facilitates perception and object recognition and can also affect functions such as maintaining balance, reading, and driving [2,3,4,5]. According to the World Health Organization (WHO), an estimated 285 million people globally are visually impacted and roughly 90% of patients are visually impaired due to chronic eye diseases, such as corneal opacities and dystrophies [6,7,8]. Corneal opacities alone affect more than 4.2 million people worldwide, making corneal diseases the fifth leading cause of visual impairment [8,9]. Those with substantial corneal opacities frequently require corneal transplantation or penetrating keratoplasty/PK [10,11,12,13], a surgical procedure that replaces the patient’s cornea with a complete or partial donor corneal tissue graft [9,13]. Although it is widely regarded as the most common and effective treatment for severe corneal injuries, approximately 20% of patients experience post-surgical complications including disease re-occurrences and graft rejections [11,12,13,14,15]. Furthermore, because of the scarcity of donor corneas, only about 1 in 70 individuals globally who require corneal transplants actually receive the procedure [11,16].

The cornea, often called the “window” of the eye, is the transparent outer layer of the ocular anterior segment, responsible for protecting and refracting light as it enters the eye [17,18,19]. It consists of five distinct layers: the epithelium, Bowman’s layer, stroma, Descemet’s membrane, and endothelium. Together, these layers contribute significantly to the eye’s refractive power [13,20,21]. The collagen-rich stroma makes up about 90% of the cornea’s thickness, and is essential for maintaining corneal strength and transparency [17,20]. Corneal integrity and transparency are vital for clear vision, and any disruptions due to injury or disease can lead to significant visual impairments or even blindness [8,9,22,23,24,25,26,27,28,29].

In corneal wound healing, the resident corneal keratocytes differentiate into myofibroblasts prior to apoptosis [20,27,30,31]. Additionally, the development of inflammation and the increased numbers of myofibroblasts have been linked to increased stromal stiffness after corneal trauma [27]. When the myofibroblast proliferates and migrates into an open wound, excessive extracellular matrix (ECM) leads to corneal scarring/fibrosis [18,32]. Alterations in the quantity and quality of the collagen due to the excessive ECM production negatively impacts corneal tissue [33,34]. Thus, an undisturbed collagenous ECM is essential to maintain the cornea’s transparency as well as its curvature [27].

Three-dimensional (3D) bioprinting is a cost-effective method for creating artificial organs and tissues [9,17,35,36,37,38,39,40,41,42,43]. While studies on 3D bioprinting date back to the late 1990s, there has been a notable surge in publications in the past five years [17,38,39,41,44,45,46,47,48]. This technology often employs automated processes and computer-aided designs (CADs) to fabricate tissues with or without living cells. It allows for the precise, adjustable, and repeatable creation of tissue models [42,49]. Beyond organ fabrication, 3D bioprinting is also used in drug delivery and personalized medicine solutions [39,40,50,51]. In ophthalmology, the biocompatible materials used in 3D bioprinting show promise towards the development of transplantable tissues with lower rejection risks [9,52].

Extrusion-based techniques have revolutionized the field of corneal bioprinting. Gingras et al. [53] developed a transparent acellular corneal model using various bioinks including collagen, alginate, and alginate-gelatin composites. Others have made significant strides by bioprinting a cornea with corneal stromal keratocytes using a collagen-based bioink and demonstrating cell viability for up to a week [17,36]. Similarly, researchers used a hyaluronic acid bioink to successfully bioprint a corneal model using human corneal endothelium-derived pluripotent stem cells and maintained cell viability for 10 days [54]. Collectively, these studies highlight the significant advancements in developing detailed and functional 3D corneal constructs [37,46,53,54,55,56,57]. However, a significant limitation to these studies is the unknown long-term viability and stability of the models. Although 3D bioprinting is still in its early stages and faces technological, ethical, and regulatory challenges [9,17,52,58], progress is being made. Innovations, particularly in multi-material 3D bioprinting techniques [59] are enhancing the ability to create affordable corneal bioprinting scaffolds [35,45,53,59].

The main objective of this study was to develop and characterize fully customizable corneal bioprinted substitutes as a potential alternative to donor corneas. This is critical so that we can ultimately provide personalized treatment, reduce reliance on donor corneas, and reduce risk of complications. To the best of our knowledge, our study is the first to demonstrate the effective use of collagen bioink combined with human corneal stromal fibroblast to create customizable synthetic corneal substitutes.

## 2. Materials and Methods

### 2.1. Ethical Information

All research conducted adhered to the tenets of the Declaration of Helsinki. Cadaveric human corneas were obtained from the National Disease Research Interchange (NDRI; Philadelphia, PA, USA). All studies and protocols were approved by the North Texas Regional Institutional Review Board (IRB #2020-030).

### 2.2. Bioprinting Setup

To support the corneal constructs, Polydimethylsiloxane (PDMS) rings were created using a 4:1 weight/weight ratio of SE 1700 (Catalog:NC1466152; Fisher Scientific, Hampton, NH, USA) and Sylgard 184 (Catalog:NC9285739; Fisher Scientific, Hampton, NH, USA). Prior to mixing the PDMS components, a 10:1 ratio of base to curing agent was prepared for each PDMS. The PDMS mixture was then transferred into a clean 10 cc syringe barrel (Advanced Solutions Life Science, Louisville, KY, USA) equipped with pistons and 27GA conical needle (0.008”; Nordson EFD, Westlake, OH, USA).

Printing was accomplished through an extrusion-based technique using the BioAssemblyBot^®^ 200 (BAB200; Advanced Solutions Life Science, Louisville, KY, USA) and the BAB Hand ambient tool. PDMS rings were designed with the aid of the Tissue Structure Information Modeling (TSIM version 1.1.232); Advanced Solutions Life Science, Louisville, KY, USA) software, a 3D digital design tool. The rings were designed with a central area of 0.52 mm and a peripheral width of 0.67 mm. The dimensions included a base diameter of 7.98 mm and a curvature radius of 4.24 mm. To ensure accurate dimensions, alignment with the x, y, z coordinates was performed using the BAB200. The rings were then printed onto a sterile plastic lid (CORNING, Corning, GA, USA) at room temperature (24 °C). The printing settings were adjusted to a line width of 0.4 mm, line height of 0.3 mm, a speed of 9 mm/s, a start delay of 25 ms, and a printing pressure of 47 PSI. To prevent the PDMS mixture from hardening, the printing pressure was gradually increased by 2–3 PSI every 30 min following the loading of the mixture into the barrel. New needle tips were used for each print to avoid clogging.

**Figure 1:**
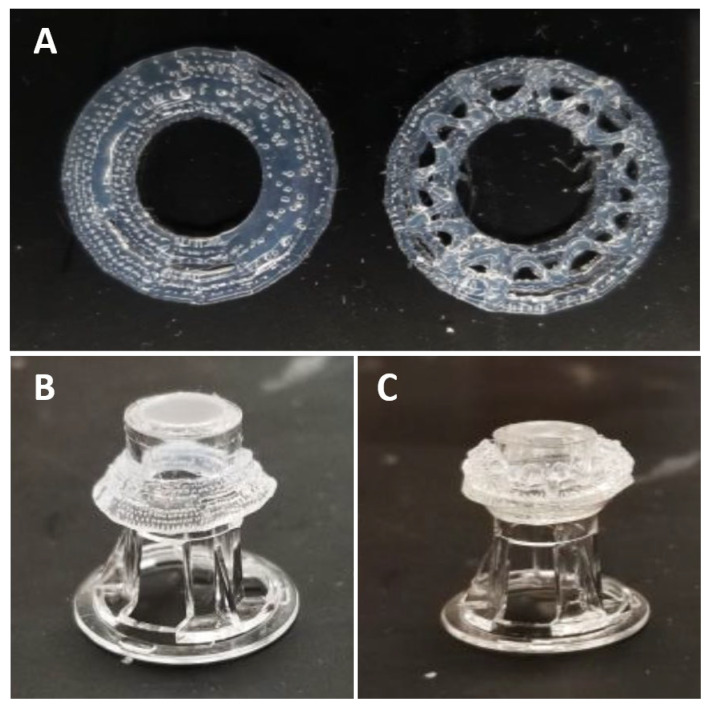
Construction of the PDMS ring. (**A**) Two bioprinted rings used to develop the PDMS rings structure. (**B**) Base layer of the PDMS rings [1st PDMS ring] fitted on a 24-well transwell insert. (**C**) 2nd PDMS ring stacked on-top of each other and fitted on a 24-well transwell insert.

All PDMS rings were incubated at 60 °C for 2 to 2 ½ h to ensure the PDMS rings were fully cured as shown in Figure 1A. They were then detached and transferred onto a glass slide. Using a Harrick Plasma PDC-001 plasma cleaner pump (Harrick Plasma, Ithaca, NY, USA), the glass slides were placed inside the chamber and allowed the pressure to reach approximately 210 TORR for one minute. Using forceps, the PDMS rings were transferred onto a 24-well transwell insert (PDMS constructs; CORNING, Corning, NY, USA) as shown in Figure 1B,C, followed by a 1-h UV light exposure for sterilization. The PDMS rings were positioned to form a small compartment between the rings when transferred onto the insert. This allows the collagen to encase between the PDMS ring.

### 2.3. Gelatin Dome

**Figure 2:**
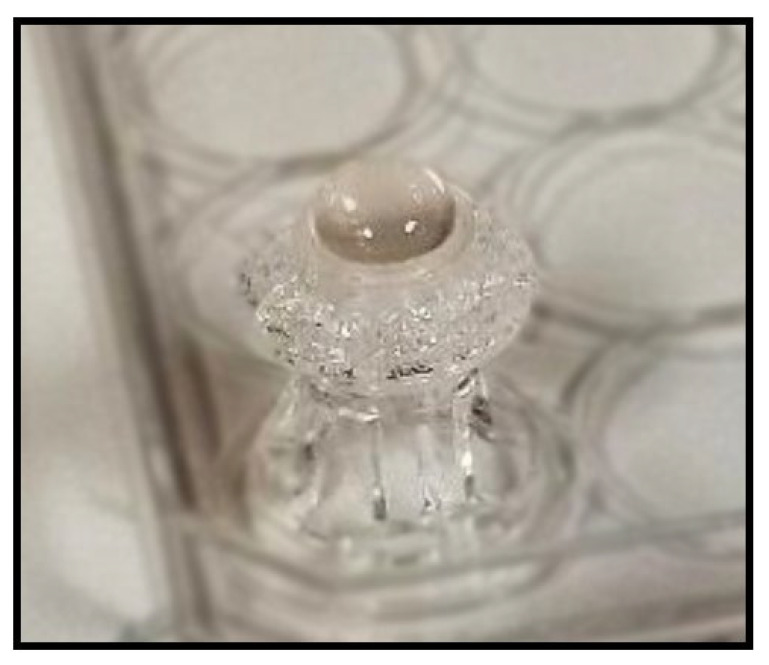
Gelatin dome on the base of the transwell insert.

In order to create the dome structure of the corneal constructs, a bead-like dome was prepared using a 6% gelatin solution. This was prepared by dissolving 1.2 g of gelatin (Sigma; St. Louis, MO, USA) in 20 mL of Phosphate Buffer Saline (PBS; ThermoFisher Scientific, Waltham, MA, USA), and heating the mixture at 65.5 °C for 30 min until it dissolved. The gelatin solution was then sterile filtered and transferred into a 30-cc syringe barrel (Advanced Solutions Life Science, Louisville, KY, USA). Using the BioAssemblyBot^®^ Hot tool (Advanced Solutions Life Science, Louisville, KY, USA), preheated to 24 °C, gelatin was added and allowed to equilibrate in the hot tool for 1 h before printing. The dispensing of warmed gelatin onto the PDMS constructs creates a bead-like dome to match the desired cornea shape as displayed in Figure 2. The constructs were incubated at 4 °C for 5 min before applying the collagen on top of the gelatin dome.

### 2.4. Acellular Collagen Constructs

**Figure 3:**
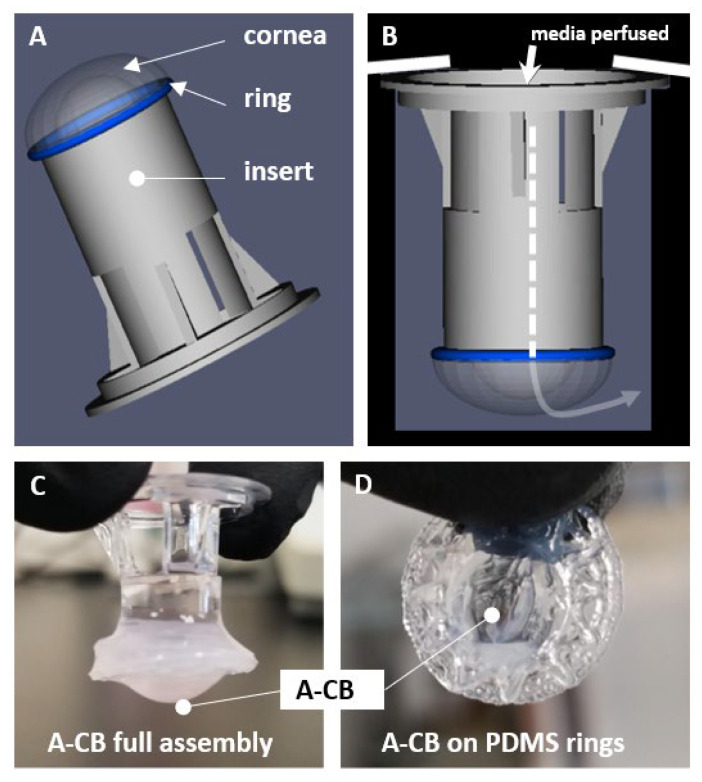
(**A**) The schematic of the placements of the A-CB, ring, and insert. with A-CBs. (**B**) The schematic of how the media would be perfused. (**C**) The gross images of the A-CBs fully assembled on a transwell insert. (**D**) The gross image of the A-CB on PDMS rings.

The acellular collagen solution was prepared at a concentration of 5 mg/mL by combining Dulbecco’s Modified Eagle Medium (DMEM), sterile ultrapure H_2_O, and a stock collagen I solution (Catalog: IKD119261001; CELLINK/Bico, Gothenburg, Sweden) following the formulation in Section 2.6.1. The DMEM was made by mixing 4 g of DMEM powder (Catalog:31600-034; Gibco, Life technologies, Carlsbad, CA, USA), 0.94 g of Sodium Bicarbonate (NaHCO3; CORNING, Corning, NY, USA), 10 mL of HEPES (Catalog: 15630-080; Gibco, Life technologies, Carlsbad, CA, USA), and 240 mL of ultrapure H_2_O. The collagen I stock solution was added last due to its temperature-sensitive nature, and all components of the mixture were kept on ice during the entire preparation process. The collagen solution was brought to a neutral pH using 1 M NaOH, kept on ice while mixing, and was used within 30 min of preparation. A volume of 200 µL was then pipetted over the top of the gelatin dome, creating a thin layer of collagen over the dome and encasing the PDMS rings as shown in Figure 3A–D. The acellular collagen bioprinted constructs (A-CBs) were placed in a humidified incubator at 37 °C with 5% CO_2_ for 30 min to allow the collagen to fully gel. A-CBs were then flipped carefully and submerged into a 24-well plate containing 1 mL of PBS and incubated.

#### 2.4.1. Water Retention

To compare water retention rates, five healthy human corneas were evaluated alongside our A-CBs. The healthy corneas were rehydrated in PBS at room temperature for 1 h. Both the A-CBs and healthy corneas were then cut into 2 × 2 mm pieces and weighed individually. Each piece was placed in a Fisherbrand Acrylic Desiccator cabinet (ThermoFisher Scientific, Waltham, MA, USA) for 30-min intervals. After each incubation, the pieces were weighed and recorded. This process was repeated for 2–3 h until the weight stabilized, indicating a plateau in water retention.

#### 2.4.2. Reflection Confocal Microscopy

The A-CBs were imaged using reflection confocal microscopy (RCM) in order to visualize the bioprinted cornea. Following 24 h of incubation at 37 °C in a humidified chamber, the samples were fixed with 2% neutral buffered formalin. Following RCM imaging, measurements of the constructs were taken at the base, periphery, and dome. The imaging was performed using an Olympus FVMPE microscope (Evident Scientific, Inc.; Waltham, MA, USA).

### 2.5. Cell Culture

Primary human corneal fibroblasts (HCFs) were isolated from healthy donor corneal rims, as previously described [18,60]. Briefly, the epithelium and the endothelium were removed with a razor blade, leaving behind the stromal layer. The corneal stromal explants were then cut into roughly 2 × 2 mm pieces and placed in a T25 flask. It was allowed to adhere for 45 min at 37 °C with 5% CO_2_ before adding complete media (RM; Eagle’s Minimum Essential Medium (EMEM; CORNING, Corning, NY, USA)) containing 10% fetal bovine serum (FBS; Atlanta Biologicals; Flowery Branch, GA, USA) and 1% Antibiotic–Antimycotic (AA; Life Technologies; Grand Island, NY, USA). The cells were grown to 80% confluence prior to trypsinization (Gibco^®^ Trypsin-EDTA 0.05% phenol red; Life technologies, Carlsbad, CA, USA) and loaded on the collagen solution. Cell passages of 4 and 5 were used for this experiment.

### 2.6. Cell-Embedded Collagen Construct Model

A preliminary series of cell concentrations was conducted using 7.5 × 10^4^ (75 K), 1.0 × 10^5^ (100 K), 1.5 × 10^5^ (150 K), and 2.0 × 10^5^ (200 K) HCFs to identify the optimal cell densities for collagen constructs over 1, 2, and 4 weeks. The 75 K and 150 K cell densities were selected as the optimal densities, as measured by microscopic evaluation and the absence of construct contraction. The cells were centrifuged at 1000 RPM for 5 min to create a pellet, the RM was removed, and the cells were resuspended in DMEM. Using the formula in Section 2.6.1, a collagen solution was prepared. The cells were added to the collagen solution and NaOH was added slowly in order to establish the correct pH level (~pH 7.4). Afterwards, 200 µL of the collagen/cell solution was pipetted over the top of the gelatin dome. The HCF-embedded corneal bioprinted constructs (3D-hCBs) were placed in a humidified incubator at 37 °C with 5% CO_2_ for 30 min to allow the collagen to fully gel. Next, the 3D-hCBs were flipped carefully and submerged into a 24-well plate containing 1 mL of RM, allowing the media to perfuse the entire construct as shown in Figure 3B. Next, they were placed back into the 37 °C with 5% CO_2_ incubator, and fresh RM was changed every other day. Following 1, 2, and 4 weeks in culture, RNA was extracted from the 3D-hCBs and quantitative Real-Time PCR (qRT-PCR) was performed. All experimental conditions were repeated three times.

#### 2.6.1. Collagen Solution Formulation

Collagen was diluted to 6 mg/mL in ultrapure H_2_O and DMEM. The pH of the collagen solution was neutralized.

#### 2.6.2. RNA Extraction

The bioprinted corneas were washed using PBS and transferred into a 1.5 mL tube containing 1 mL of Ambion TRIzol (Ambion TRIzol^®^; Life technologies, Carlsbad, CA, USA). The collagen was then homogenized using a Fisherbrand^TM^ 150 Hand Held Homogenizer (Fisher Scientific; Hampton, NH, USA) on ice for 1 min, followed by 3-min incubation on ice, and a second 1-min homogenization on ice. RNA extraction was then carried out according to the previously described protocol [61]. An ultraviolet spectrometer (Epoch 2; BioTek Instruments Inc, Agilent, Santa Clara, CA, USA) was used to measure the quantity and purity of the extracted total RNA.

#### 2.6.3. qRT-PCR

RNA to cDNA synthesis was performed using SuperScript III First-Strand Synthesis SuperMix (Invitrogen, Carlsbad, CA, USA) per manufacturer’s protocol. qRT-PCR was performed using TaqMan gene expression assay (Applied Biosystems, Life Technologies; Foster City, CA, USA). The following PCR probes (ThermoFisher Scientific; Rockford, IL, USA) were tested: GAPDH (Catalog: Hs99999905_m1) and 18s (Catalog: Hs99999901_s1) as housekeeping, Collagen I ([COL I] Catalog: Hs00164004_m1), Collagen III ([COL III] Catalog: Hs00943809_m1), Collagen V ([COL V] Catalog: Hs00609133_m1), αSmooth Muscle Actin ([αSMA] Catalog: Hs00426835_g1), Cellular Fibronectin ([cFN] Catalog: Hs00365052_m1), Thrombospondin-1 ([THBS1/TSP-1] Catalog: Hs00962908_m1), Vimentin ([VIM] Catalog: Hs00185584_m1), and Vinculin ([VCL] Catalog: Hs00419715_m1). Furthermore, a mixture of TaqMan Fast Advanced Master Mix (Applied Biosystems, Life Technologies; Foster City, CA, USA), desired probes, and 10 ng of cDNA was used for the PCR reaction.

### 2.7. Statistical Analysis

Statistical analysis was conducted using GraphPad Prism 10.2.0 software and Microsoft Excel (MS-Excel). One-way ANOVA analysis was performed where *p* < 0.05 was considered statistically significant.

## 3. Results

### 3.1. 3D Bioprinted Human Corneal Model

**Figure 4:**
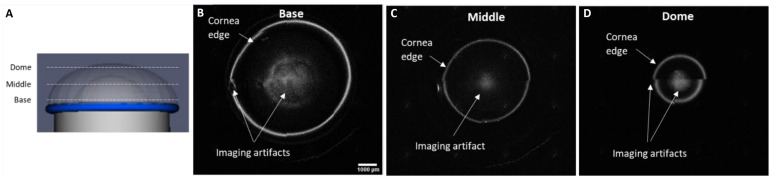
(**A**) The schematic figure of how different layers of the RCM are labeled. (**B**) The base radial sections of the RCM image of 3D bioprinted cornea construct/A-CB on top of the transwell. (**C**) The middle radial sections of the RCM image of 3D bioprinted cornea construct/A-CB on top of the transwell. (**D**) The dome radial sections of the RCM image of 3D bioprinted cornea construct/A-CB on top of the transwell.

Using physical dimensions of the human cornea, we developed a method to fabricate cornea equivalents that (1) maintained the native cornea structure, (2) allowed controllable size and shape (i.e., human cornea size), and (3) was compatible with cell culture methods supporting cellularization as the application required. Figure 4A explores the schematic of how each layer was imaged through RCM. Figure 4B–D demonstrates RCM images of a concentric dome of thin collagen extending from the base of the transwell to the dome peak. The fabricated tissue model measured at a thickness of 0.52 mm at the central area, 0.67 mm at its periphery, and its dimensions measuring 7.98 mm at its base with a 4.24 mm radius of curvature. These dimensions were similar to the thickness of an adult human cornea which ranges between 420 and 625 µm in the center and 633 and 673 µm in the periphery on average [62,63,64].

### 3.2. Acellular Model Results (A-CB)

#### Observation of Water Retention

**Figure 5:**
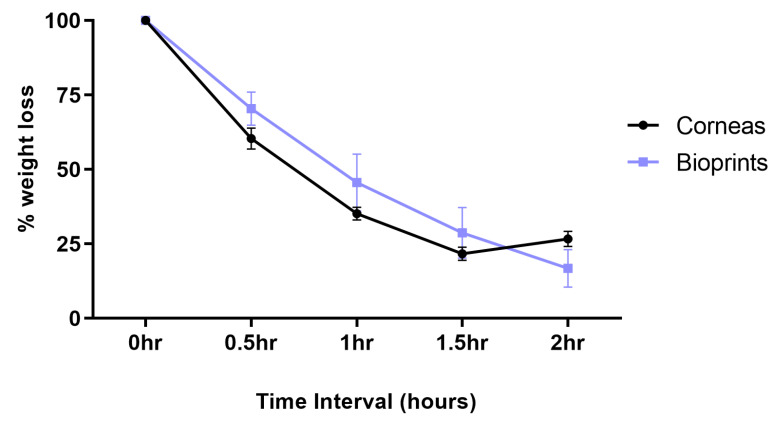
Water retention analysis of human corneas (*n* = 5) and A-CBs (*n* = 4) at time intervals 0, 0.5, 1, 1.5, and 2 h. Statistical analysis was performed using 2way ANOVA, where no significant differences were observed.

Water retention is crucial for the maintenance of the human cornea. Without the correct water balance, swelling and corneal edema can occur. We determined the water retention rates of A-CBs and human corneas by measuring their weight after incubating in a desiccator at 30-min intervals for 2 h as shown in Figure 5. Corneal tissues and printed A-CBs steadily decreased in the weight-loss percentage up until 1.5 h and then plateaued by 2 h. We found no significant differences in the water loss percentage between A-CBs and the corneal tissues.

### 3.3. Cellular Model Results (3D-hCB)

#### 3.3.1. Collagen III (COL III), α-Smooth Muscle Actin (αSMA), and Cellular Fibronectin (cFN)

**Figure 6:**
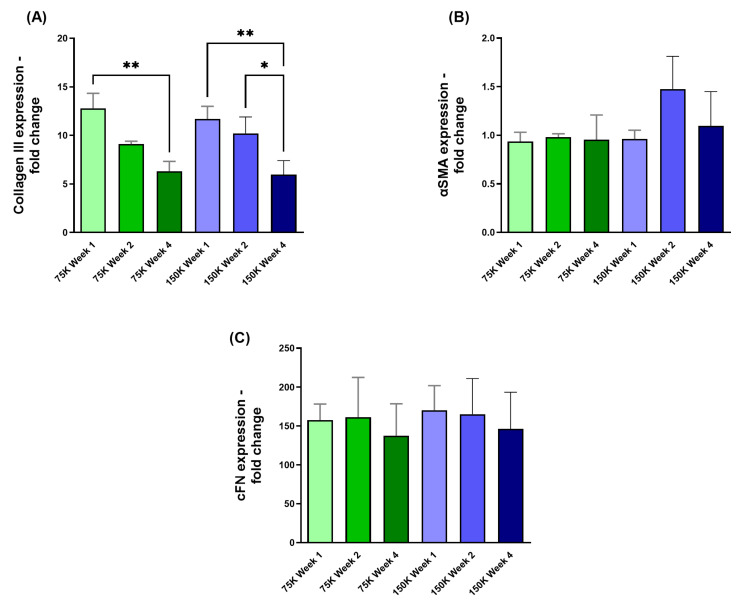
3D-hCB constructs with HCF densities of 75 K and 150 K. (**A**) COL III expression observed in 3D-hCBs constructs at week 1, 2, and 4. (**B**) αSMA expression observed in 3D-hCBs at timepoints week 1, 2, and 4. (**C**) cFN expression observed at week 1, 2, and 4 for both HCF densities. * = *p* < 0.05; ** = *p* < 0.01. *n* = 3.

The expression levels of COL III and αSMA were analyzed using qRT-PCR for two HCF densities (75 K and 150 K) over 1-, 2-, and 4-week timepoints. Significant downregulation of COL III expression was observed in both HCF densities from week 1 vs week 4 (75 K; *p* = 0.0042, 150 K; *p* = 0.0087; Figure 6A). Additionally, the 150 K 3D-hCBs showed significant downregulation of COL III from week 2 to week 4 (150 K; *p* = 0.0394; Figure 6A). Conversely, αSMA expression (Figure 6B) and cFN expression (Figure 6C) did not exhibit longitudinal variations between the two different HCF densities.

#### 3.3.2. Collagen I (COL I) and Collagen V (COL V)

**Figure 7:**
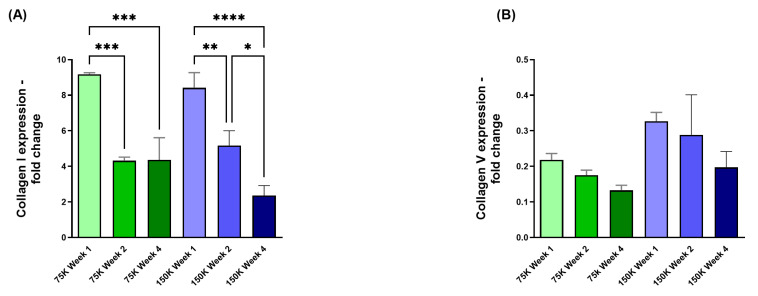
3D-hCB constructs with HCF densities of 75 K and 150 K. (**A**) COL I expression observed at week 1, 2, and 4 for both HCF densities. (**B**) COL V expression observed at week 1, 2, and 4 for both HCF densities. * = *p* < 0.05; ** = *p* < 0.01; *** = *p* < 0.001; **** = *p* < 0.0001. *n* = 3.

The overall COL I expression showed significant downregulation in both HCF densities of 75 K and 150 K. The 75 K 3D-hCBs expressed significant downregulation of COL I when comparing week 1 to weeks 2 and 4 (75 K; *p* = 0.0006, *p* = 0.0007; Figure 7A). The 150 K 3D-hCBs expressed significant downregulation of COL I when comparing week 1 to weeks 2 and 4 (150 K; *p* = 0.0098, *p* = <0.0001; Figure 7A), and in week 2 compared to week 4 (150 K; *p* = 0.021; Figure 7A). No longitudinal differences in expression of COL V (Figure 7B) were observed in either HCF densities.

#### 3.3.3. Thrombospondin-1 (TSP-1), Vimentin (VIM), and Vinculin (VCL)

**Figure 8:**
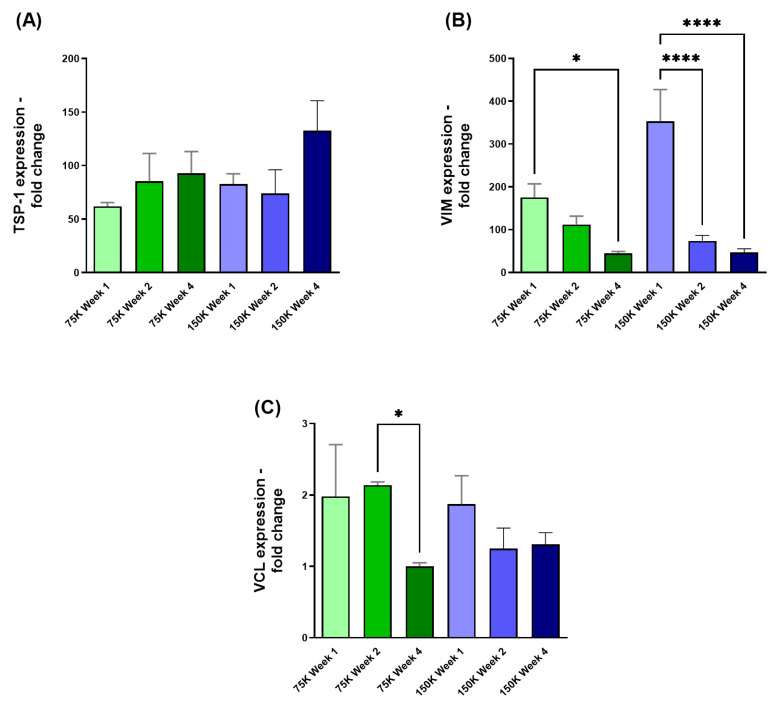
3D-hCBs with HCF densities of 75 K and 150 K. (**A**) TSP-1 expression observed at week 1, 2, and 4 for both HCF densities. (**B**) VIM expression observed at week 1, 2, and 4 for both HCF densities. (**C**) VCL expression observed at week 1, 2, and 4 for both HCF densities. * = *p* < 0.05; **** = *p* < 0.0001. *n* = 3.

The TSP-1 expression in Figure 8A was observed to have no significance over time for either HCF densities. However, significant downregulation in VIM expression was observed across both HCF densities when comparing 75 K week 1 to 75 K week 4 (75 K; *p* = 0.0204; Figure 8B), and 150 K week 1 to 150 K weeks 2 and 4 (150 K; *p* = <0.0001, *p* = <0.0001; Figure 8B). When examining the expression of VCL, significant downregulation was observed only when comparing the 75 K week 2 to 75 K week 4 (75 K; *p* = 0.048; Figure 8C).

## 4. Discussion

The emergence of 3D bioprinting technology has led to promising biological models [39,41,65,66,67,68,69,70]. Numerous scientific publications have reviewed various biocompatible materials and created human tissues models, some of which are used in clinical settings [65,67,71,72,73,74,75]. In ophthalmology, bioprinting has made it possible to produce artificial corneas and explore regenerative techniques for retinal tissue, including the printing of individual retinal layers [65,76,77,78,79,80,81]. Notably, in corneal research, 3D bioprinted corneal tissues models have been developed, utilizing decellularized frameworks to characterize these tissues [17,56,82,83,84,85]. However, replicating the cornea’s complex structure artificially is challenging due to its layered architecture, the need for transparency linked to precise collagen arrangement, and the unique microenvironment of each layer. Scaling productions, long-term stability, replicating avascularity and innervation add further complexity to this difficult endeavor [56,65,71,79].

One of the key factors in developing tissue regenerative models through 3D bioprinting is understanding cell viability, the role of ECM involvement, and cell–ECM interactions [36]. Kutlehria et al., 2020 [36] noted that the development of bioinks primarily relies on ECM-derived components like gelatin, agarose, type I collagen (COL I), fibronectin, and hyaluronic acid. Additional bioinks can also incorporate materials such as alginate, chitosan, and decellularized extracellular matrix (dECM) [36,45,86]. For instance, Isaacson et al., 2018 [17] used sodium alginate and methacrylated type I collagen in their bioinks, while Wu et al., 2016 [87] mixed gelatin/alginate solutions with neutralized rat-tail type I collagen. However, Das et al., 2015 [88] identified significant limitations with alginate-based bioinks, including a considerable loss of mechanical properties during in vitro culture and variable cell responses. Additionally, there are limited studies exploring the use of materials like sodium alginate and gelatin incorporated with cells [89,90]. Zhang et al., 2019 [89] demonstrated the use of sodium alginate-gelatin solution to provide a more controllable geometric scaling and the quality of a 3D corneal bioprinting scaffold model ideal for patients.

A lack of bioink mechanical strength, particularly for collagen-only bioinks, can be problematic, hindering structural integrity during bioprinting [91,92,93]. Collagen is a major component of the cornea, providing strength and structure to a highly organized tissue [94,95]. Collagen fibrils are considered the fundamental load-bearing components and are uniquely narrow yet strong and protective. Replicating the overall complex structure, including the seven identified fibril types [96,97], remains a significant hurdle in the field.

Collagen types I and V are essential components of a healthy human cornea, while collagen type III is associated with corneal fibrosis [30,98]. Collagen I is the most abundant type in the human cornea, followed by collagen V, with collagen III increasing primarily during wound healing [30,99]. Our data revealed a decrease in both COL I and COL III after the first week, indicating ECM remodeling. Furthermore, we investigated fibrotic markers (TSP-1, VIM, VCL) all involved in the corneal wound healing processes [100,101,102,103,104,105,106,107]. The observed significant downregulation of VIM and VCL after week 1 suggests a reduction in fibrotic activity at later time points in our model. Thus, a potential shift away from a wound-healing/fibrotic state, although further investigation is warranted to fully understand the ECM remodeling process and its implications for our model. The observed changes in collagen types and fibrotic markers provide valuable insights into the dynamics of the ECM and its potential role in successful corneal substitute development.

Our novel model exhibits promising characteristics; however, further research is warranted in order to fine tune and achieve the ultimate/desirable balanced and functional ECM that is crucial for the long-term success of any artificial cornea [71,79,108,109]. Further studies should focus on elucidating the specific mechanisms driving ECM remodeling in this model, as well as developing strategies to fine-tune this process to create corneal substitutes that closely mimic the structure and function of the native tissue. Such strategies include the investigation of growth factors, cytokines, and other signaling molecules in regulating collagen synthesis, degradation, as well as exploring the potential of geometric parameters that can promote a healthy and stable ECM environment.

## Data Availability

The original data presented in the study are openly available in FigShare at https://doi.org/10.6084/m9.figshare.28664744.

## Abbreviations

The following abbreviations are used in this manuscript:

PDMS	Polydimethylsiloxane
BAB200	BioassemblyBot^®^200
TSIM	Tissue Structure Information Modeling
A-CBs	Acellular collagen bioprinted constructs
3D-hCBs	HCF-embedded corneal bioprinted constructs
RCM	Reflection confocal microscopy
qRT-PCR	Quantitative Real-Time PCR
ECM	Extracellular Matrix
dECM	Decellularized Extracellular Matrix
